# Text Mining Approaches to Analyze Public Sentiment Changes Regarding COVID-19 Vaccines on Social Media in Korea

**DOI:** 10.3390/ijerph18126549

**Published:** 2021-06-18

**Authors:** Jae-Geum Shim, Kyoung-Ho Ryu, Sung Hyun Lee, Eun-Ah Cho, Yoon Ju Lee, Jin Hee Ahn

**Affiliations:** Department of Anesthesiology and Pain Medicine, Kangbuk Samsung Hospital, Sungkyunkwan University School of Medicine, Seoul 03181, Korea; jaegeum77@naver.com (J.-G.S.); kyoungho.ryu@hotmail.com (K.-H.R.); hoho4321.lee@hanmail.net (S.H.L.); eunah.cho@daum.net (E.-A.C.); nowyjhere@gmail.com (Y.J.L.)

**Keywords:** COVID-19 vaccines, topic modeling, sentiment analysis, Korea

## Abstract

The COVID-19 pandemic has affected the entire world, resulting in a tremendous change to people’s lifestyles. We investigated the Korean public response to COVID-19 vaccines on social media from 23 February 2021 to 22 March 2021. We collected tweets related to COVID-19 vaccines using the Korean words for “coronavirus” and “vaccines” as keywords. A topic analysis was performed to interpret and classify the tweets, and a sentiment analysis was conducted to analyze public emotions displayed within the retrieved tweets. Out of a total of 13,414 tweets, 3509 were analyzed after preprocessing. Eight topics were extracted using the Latent Dirichlet Allocation model, and the most frequently tweeted topic was vaccine hesitation, consisting of fear, flu, safety of vaccination, time course, and degree of symptoms. The sentiment analysis revealed a similar ratio of positive and negative tweets immediately before and after the commencement of vaccinations, but negative tweets were prominent after the increase in the number of confirmed COVID-19 cases. The public’s anticipation, disappointment, and fear regarding vaccinations are considered to be reflected in the tweets. However, long-term trend analysis will be needed in the future.

## 1. Background

Coronavirus disease 19 (COVID-19) was first reported in Wuhan, China, at the end of 2019, and has rapidly spread worldwide since then [1,2]. There are currently more than 120 million confirmed cases of COVID-19 worldwide and more than 100,000 cases within Korea. There is no definitive way to prevent COVID-19 infection other than basic measures, such as wearing a mask, washing one’s hands, and social distancing. The most effective way to deal with the COVID-19 pandemic is the fast and successful development of COVID-19 vaccines. The prevention rate of COVID-19 vaccines is over 80 percent, and they are expected to be a game-changer [3,4]. However, anxiety and distrust about the vaccine’s effectiveness and safety have become major obstacles to rapid vaccination [5,6].

Recently, the Internet has become the primary source of information about health and vaccination for most people [7]. Twitter is an extremely popular social media platform because of its rapid information dissemination and its application in various domains such as business, disaster recovery, and healthcare. Social media users express their opinions and emotions related to COVID-19 issues through Twitter [8].

Text mining is a field of data mining that identifies meaningful patterns and information from large databases [9]. Currently, text mining on social media is being applied to analyze public opinion on or responses to the COVID-19 pandemic [10,11]. Data mining and text analysis have also been used to study the attitudes of vaccine deniers and their reasons for rejecting vaccines [12]. Korea commenced COVID-19 vaccinations on 26 February 2021. The public response to this was apparent on Twitter [13].

Therefore, we aimed to investigate the Korean public response to COVID-19 vaccines on Twitter from 23 February 2021 to 22 March 2021. We focus on this issue in the context of the recent commencement of vaccinations against COVID-19, and explore the arguments and emotions present in relevant tweets. We collected tweets related to COVID-19 vaccines on Twitter and implemented topic modeling to extract and classify topics. Then, a sentiment analysis using the KNU sentiment lexicon was performed [14].

## 2. Materials and Methods

### 2.1. Data Source and Preprocessing

We collected tweets to build our own Twitter data crawler using an open-source library called “rtweet” [15]. Our investigation focused on South Korea. Using the Korean words for “coronavirus” and “vaccine” as keywords, tweets posted from 23 February 2021 to 22 March 2021 were extracted for our analysis. This study collected the following tweet information: user ID, Twitter ID, time of creation of tweet, and text content. A total of 13,414 tweets were retrieved.

**Figure 1:**
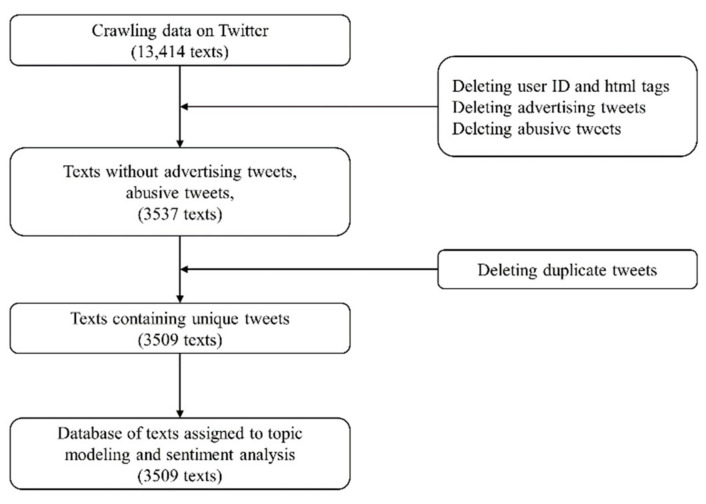
Schematic of dataset gathering and preprocessing.

The raw Twitter text data contained unnecessary information such as user ID tags and html tags, which increases the complexity of the analysis. Therefore, the text data were preprocessed to obtain a clean and analyzable dataset, as shown in Figure 1. First, the user IDs and html tags were removed. Secondly, users who posted an extreme number of tweets reflected only a few opinions; therefore, we restricted contributions to five tweets per day per user. In addition, duplicate tweets were removed because sometimes one user or multiple users repeatedly posted the same tweet. After filtering and removing unnecessary text, only 3509 tweets were retained for the analysis.

### 2.2. Method

Information on the number of confirmed COVID-19 cases was obtained using the open application program interface (API) in the public data portal operated by the Korean government [16]. In addition, to obtain tweets regarding COVID-19 vaccines, the Twitter API was accessed using a developer account.

**Figure 2:**
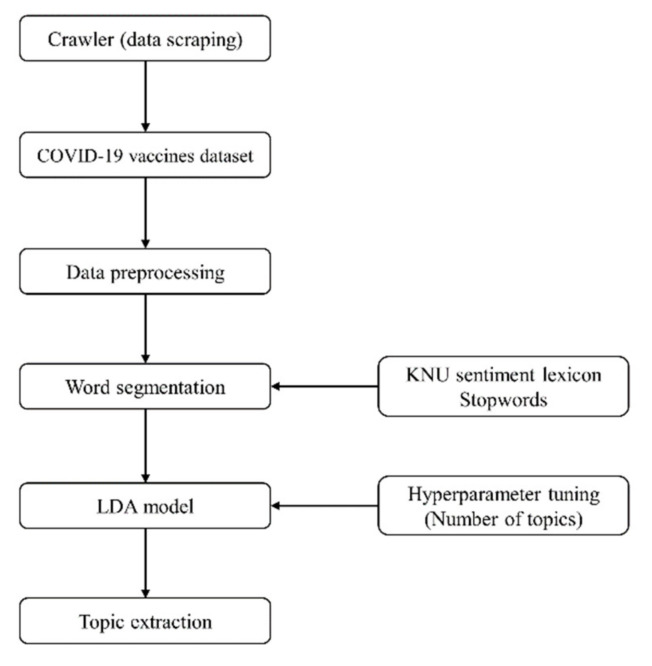
The process of topic extraction.

To explore the public response to COVID-19 vaccines, we performed a correlation analysis between the number of new confirmed cases and the number of tweets regarding COVID-19 vaccines, topic modeling, and sentimental analysis. Regular expressions were used to search and replace the matched string of text using text editors in R software. In our study, we report topic extraction from a corpus of tweets using a probabilistic topic model––the Latent Dirichlet Allocation (LDA) model. The topic modeling process conducted in this study is outlined in Figure 2.

#### 2.2.1. Topic Modeling and LDA

Topic modeling is a method of dividing collections of documents into natural groups to understand them separately. LDA is the most widely used method for fitting topic models [17,18]. It is a three-level hierarchical generative model that mirrors the typical use of natural language [19]. Through the following assumptions in the LDA model, the relationship between topics and words is estimated. A topic consists of a mixture of words; a document consists of a mixture of topics. Thus, multiple words are included in one topic with different probabilities and the same word in multiple topics with different probabilities [20]. LDA was used to classify text in COVID-19 vaccine-related tweets to a particular topic. To implement the LDA model, we created a Document-Term Matrix (DTM) which was analyzed in the topicmodels package in R software. A DTM is a mathematical matrix that describes the frequency of terms (or words) in the corpus (or a collection of documents). In a DTM, documents in the collection are represented by rows and terms by columns. To create a DTM, we gathered the word frequency per document. Eight topics were extracted from the raw text through hyperparameter tuning, which was proposed by Griffiths and Steyvers [20]. We used the LDA function from the topicmodels package to create an eight-topic LDA model. LDA functions and parameters from the topicmodels package were set with k and method. k is an integer representing the number of topics in the model. We used a k value of eight in our LDA model. The method is a sampling technique to be used for fitting. The iterative process to estimate the distribution of the topic and words was implemented using a technique called Gibbs sampling. Table 1 presents the COVID-19 vaccine-related topics and keywords.

#### 2.2.2. Sentiment Analysis

Sentiment analysis is used to analyze the emotions or opinions contained in text. It can help develop understandings of the feelings embedded in a writer’s text and about which topics people feel positive or negative. It is a natural language processing technique applied to determine whether a dataset contains neutral, positive, or negative semantics.

In the current study, sentiment analysis was conducted by applying the KNU Korean sentiment lexicon extracted from a sentiment classification model based on bidirectional long/short-term memory (Bi-LSTM) [21]. The KNU Korean Sentiment Lexicon is a general-purpose dictionary based on the Korean language and is more advanced than existing general-purpose lexicons. It consists of sentiment vocabularies obtained by analyzing the glosses contained in the Standard Korean Language Dictionary. The Bi-LSTM model is a neural network architecture that automatically determines the emotional polarity of each word and phrase extracted from the glosses classified as positive or negative.

Specifically, the sentiment lexicon contains a total of 14,854 sentiment vocabularies that express feelings and numbers that express the intensity of emotions, each of which has a polarity of −2, −1, 0, 1, or 2. The closer the outcome value is to 2, the more positive the emotions are, and the closer it is to −2, the more negative the emotions are. Using a sentiment lexicon, we can assign sentiment scores to words in a sentence and add them together to understand what emotions are expressed in a sentence. All the retrieved tweets were categorized as either positive, negative, or neutral to determine the frequency, and then the relative weights of the emotion categories of the tweets were applied. The sentiment scores are classified as follows: positive: score ≥1; neutral: −1 < score < 1; and negative: score ≤−1.

### 2.3. Statistical Analysis

Descriptive statistics were used to analyze the retrieved tweets. The correlation between the number of tweets regarding COVID-19 vaccines and new COVID-19 confirmed cases was analyzed through a Spearman’s correlation analysis. Statistical significance was set at *p* < 0.05.

## 3. Results

**Figure 3:**
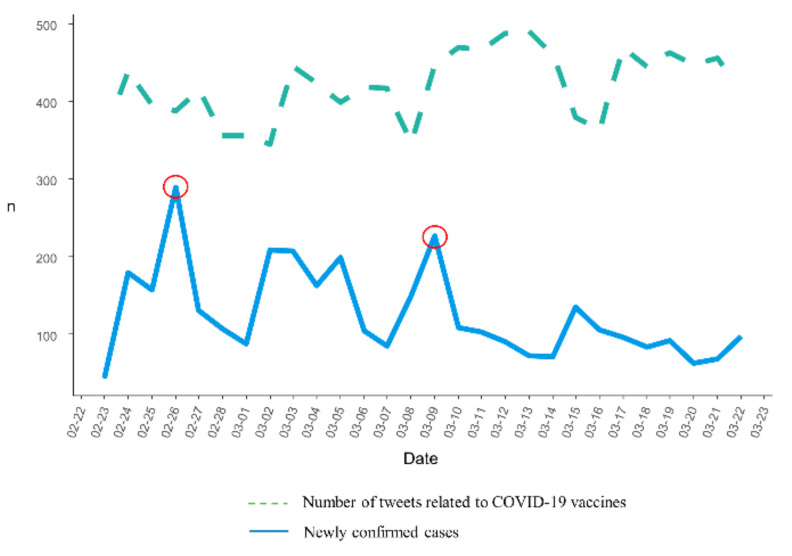
The trend of the number of tweets related to COVID-19 vaccines and newly confirmed cases.

A total of 13,414 tweets posted during the study period were retrieved. After preprocessing the raw data, 3509 tweets were used for the analysis. The trend of the number of tweets that referred to COVID-19 vaccines and the number of newly confirmed cases from 23 February 2021 to 22 March 2021, is shown in Figure 3. The number of daily COVID-19 confirmed cases in Korea was 344 to 490 during the study period. Figure 3 shows the trend of the number of newly confirmed COVID-19 patients and the number of tweets related to the COVID-19 vaccine. However, there was no significant correlation between the number of tweets about the COVID-19 vaccine and the number of confirmed COVID-19 cases (correlation coefficient = −0.354, *p* = 0.06). The curve shows two peaks, on 26 February and 9 March, in terms of the number of tweets.

### 3.1. Topic Analysis

Table 1 presents the COVID-19 vaccine-related topics and keywords. Among all topics, “vaccine hesitancy” was the most frequent topic with a 14.2% relative weight. The second and third most frequent topics were “development of vaccine” (13.1%) and “quarantine prevention policy” (13.0%). These were followed by “efficacy of vaccination” (12.6%), “priority vaccination of hospital workers” (12.0%), “media on COVID-19 vaccines” (11.9%), “medical association’s response” (11.8%), and “adverse reactions” (11.4%).

### 3.2. Sentiment Analysis

**Figure 4:**
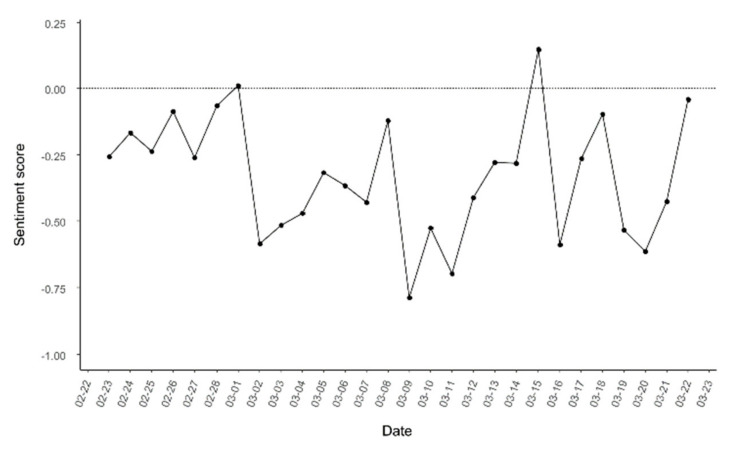
The trend of daily sentiment scores.

We conducted sentiment analysis on 3509 tweets before and after the commencement of the COVID-19 vaccinations in Korea to investigate the public response to it. The distribution of sentiment scores was between −16 and 16 for each tweet. Figure 4 shows the mean sentiment scores for all daily tweets during the study period. Correlation between the sentiment score and the number of newly confirmed cases was r = −0.430 (*p* = 0.02).

**Figure 5:**
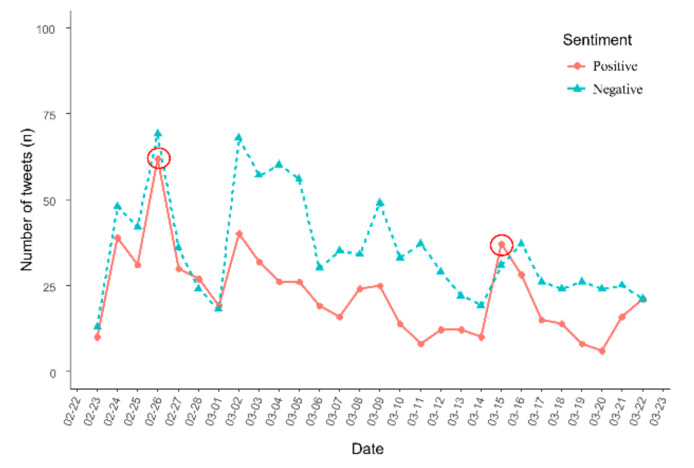
The trend of the number of positive and negative tweets.

Figure 5 shows the number of positive and negative tweets that were retrieved. The number of tweets with positive sentiment and tweets with negative sentiment tended to increase before the commencement of vaccinations on 26 February, and both displayed a decreasing tendency after vaccination. Then, from 1 March, as the number of confirmed cases of COVID-19 increased again, the number of negative tweets increased in comparison to the number of positive tweets. From 13 to 15 March, the number of new COVID-19 cases decreased by about 23%. On 15 March, the number of positive tweets temporarily increased in comparison to other days. The tweets posted on 15 March were related to keywords such as “relief” and “experience”.

## 4. Discussion

In this paper, we conducted an analysis of the Korean public response to COVID-19 vaccines by examining the topics and sentiments present within tweets regarding COVID-19 vaccine that were posted from 21 February 2021 until 22 March 2021. Healthcare workers were prioritized to receive the vaccination. Two types of vaccines were introduced in Korea: the AstraZeneca and the Pfizer vaccines. Vaccination had not yet started for the general public in this period, and the efficacy and safety of the AstraZeneca vaccine, which accounted for the largest number of vaccines secured by the government, were reported daily in the media. This is the first study to analyze the content of Korean tweets related to COVID-19. The most prominent topic was “vaccine hesitancy,” which consisted of words such as fear, flu, injection pain, time course, and degree of symptoms. The sentiment analysis revealed a similar ratio of positive and negative tweets immediately before and after the commencement of vaccinations, but negative tweets were more prevalent after the increase in the number of confirmed COVID-19 cases.

Text mining has frequently been used to analyze public opinion on or responses to the COVID-19 pandemic [10,11,22]. Sentiments, information, opinions, etc., are expressed in tweets posted by users. On 26 February, people’s positive expectations may have been reflected in their tweets as vaccinations began for the first time in Korea. The most tweeted words on 15 March were “relief” and “experience”. Lee et al. examined public opinion regarding the COVID-19 pandemic immediately after its proliferation in South Korea; during that time, the most tweeted emotional words were “overcome” and “support,” and the economic downturn appeared to be the primary topic of concern [22]. In addition, in the United Kingdom, the main topics were the government audit and healthcare workers, and in the United States, the global COVID-19 pandemic was the most discussed topic [23]. The number of confirmed cases, quarantine policies, and degree of vaccine security in each country are different; therefore, public responses and interests are inevitably varied as well. Twitter is one of the easiest tools with which the public can express their opinion, and it is an equally useful tool for analyzing such public opinion.

Our results showed no significant correlation between the number of confirmed cases and the number of tweets. The total number of daily tweets increased on days when there were prominent vaccine-related social issues. The most tweeted key phrase on February 26 was “Korean medical association.” On that day, the Korean Medical Association issued an announcement suggesting that it may not cooperate with the government’s measures to combat COVID-19 in protest to new legislation. On March 9, “special syringes to vaccinate against COVID-19” was the most frequently tweeted phrase. The Korea Disease Control and Prevention Agency (KDCA) announced a special type of syringe that could minimize vaccine waste by reducing the space between the needle and plunger. The use of this special syringe can increase the number of vaccine recipients to one or two per bottle of vaccine. As such, the number of tweets was affected by the issue that dominated the day, but was not related to the number of confirmed COVID-19 cases. However, the sentiment score of the tweets decreased as the number of confirmed COVID-19 cases increased, which indicated an increase in the number of negative tweets. In a comparative study of the public response to COVID-19 in the United Kingdom and the United States on Twitter, the sentiment score of tweets from the United Kingdom, which had fewer new confirmed cases during the study period, was higher than that of the United States [23]. This indicates that the increase in the number of confirmed cases is linked to negative emotions in at least a certain number of tweets, although it may not lead to an increase in the number of tweets about COVID-19 vaccines.

Vaccine hesitancy, vaccine efficacy, and adverse reactions accounted for a large portion of the topics that were discussed in the retrieved tweets. This seems to be related to global concerns about the side effects of the AstraZeneca vaccine which is being primarily used in Korea [24]. The AstraZeneca vaccine is more controversial in comparison to those from Pfizer and Moderna, because of its lack of clinical results and side effects such as thrombosis. Thus, it can be interpreted that public concern about this vaccine manifested on Twitter [25,26,27]. The lack of scientific trust in vaccines can result in the proliferation of conspiracy theories or misinformation on Twitter [10,11]. Given that the Korean vaccination drive is currently in its early stages, with less than 2% of the population receiving a vaccine, this study can help predict social responses or problems in advance once vaccinations commence on a larger scale. 

Our study has several limitations. First, because our research was limited to Twitter, it cannot be generalized to reflect the opinion of the entire Korean populace. If additional analyses were conducted on Facebook or Korea’s main portal sites (e.g., Naver and DAUM), these may facilitate the acquisition of a more comprehensive public response. Secondly, most Twitter users had posted tweets based on indirect experiences via the Internet and news rather than direct experiences with vaccines. This is because during the study period, vaccination began with only a small number of people, including healthcare workers. Another meaningful result could be derived by examining the change in the sentimental score of the public after vaccination. Thirdly, from a methodological perspective, the relatively small number of tweets that were retrieved may not comprehensively reflect public opinion. Thus, further in-depth examination is needed in the future.

## 5. Conclusions

The main topic of the tweets was “vaccine hesitancy”, which reflects concerns about the safety of vaccines. The ratio of positive and negative tweets were similar immediately before and after the commencement of vaccinations, but negative tweets were prominent after the increase in the number of confirmed COVID-19 cases. The public’s anticipation, disappointment, and fear regarding the vaccine were reflected in the tweets. However, long-term trend analysis is needed in the future. 

## Figures and Tables

**Table 1:** Topics related to COVID-19 vaccines.

	Topic Name	Relative Weight (%)	LDA Keywords
1	Vaccine hesitancy	14.2	Fear, flu, safety of vaccination, time course, degree of symptoms
2	Development of vaccine	13.1	Korea, United States, development, president, situation
3	Quarantine prevention policy	13.0	Mask, government, people, quarantine prevention, today
4	Efficacy of vaccination	12.6	AstraZeneca, virus, confirmed case, effect, vaccination
5	Priority vaccination of hospital workers	12.0	Adverse effect, hospital, patient, problem, flu vaccine, vaccination priority
6	Media on COVID-19 vaccines	11.9	News, start, Japan, bad journalist *, herd immunity, response
7	Medical association’s response	11.8	Nation, doctor, Korea, human, refusal, article
8	Adverse reactions	11.4	Death, adverse, prevention, infection, underlying disease

* a compound word consisting of the words “journalist” and “trash” is used in Korea.

## Abbreviations

API	application program interface
LDA	Latent Dirichlet Allocation
Bi-LSTM	bidirectional long/short-term memory
KDCA	Korea Disease Control and Prevention Agency
